# Role of Inflammatory Markers and Radiological Profile in Predicting Acute Pancreatitis Severity: A Prospective Analysis

**DOI:** 10.7759/cureus.89033

**Published:** 2025-07-30

**Authors:** Isha Stutee, Naresh K Midha, Monika Chaudhary, Deepak Kumar, Mithu Banerjee, Pawan Garg, Subhash Soni, Mahendra Kumar Garg

**Affiliations:** 1 General Medicine, All India Institute of Medical Sciences, Jodhpur, Jodhpur, IND; 2 Biochemistry, All India Institute of Medical Sciences, Jodhpur, Jodhpur, IND; 3 Diagnostic and Interventional Radiology, All India Institute of Medical Sciences, Jodhpur, Jodhpur, IND; 4 Surgical Gastroenterology, All India Institute of Medical Sciences, Jodhpur, Jodhpur, IND

**Keywords:** acute pancreatitis, bisap score, computed tomography severity index (ctsi), lactate dehydrogenase (ldh), prognostic biomarkers

## Abstract

Background

Acute pancreatitis (AP) is an inflammatory condition of the pancreas that varies in severity. Reliable prognostic markers are essential for early risk stratification. This study evaluates inflammatory markers and severity indices to predict disease outcomes.

Aims

This prospective observational study aims to assess the prognostic value of various inflammatory markers alongside the Bedside Index for Severity in Acute Pancreatitis (BISAP) and the Computed Tomography Severity Index (CTSI) in patients suffering from AP.

Methods

This study evaluated the prognostic significance of inflammatory markers in 80 patients with AP. Among these markers are the neutrophil-lymphocyte ratio (NLR), lymphocyte-monocyte ratio (LMR), platelet-lymphocyte ratio (PLR), red cell distribution width (RDW), C-reactive protein (CRP), interleukin-6 (IL-6), and lactate dehydrogenase (LDH). Their correlation with BISAP, CTSI, and Modified CTSI was also analyzed to enhance predictive accuracy.

Results

Out of 80 patients studied, alcohol was the most common cause, seen in 41 cases (51%). Gallstones were the next major etiology, found in 19 patients (24%). The age of participants ranged from 20 to 80 years, with a mean age of 40.9 ± 13.9 years. A clear male predominance was observed, with 60 (75%) patients being male.

In this study, 17 patients (21%) had mild, 54 (68%) moderately severe, and nine (11%) severe pancreatitis. LDH was significantly higher in severe cases (p < 0.0001), with levels above 435 U/L predicting severity with 100% sensitivity and 66.8% specificity. NLR, LMR, and PLR declined significantly during recovery (p = 0.001). Higher LDH, BISAP, CTSI, MCTSI, and urea levels were associated with higher mortality, whereas CRP, IL-6, NLR, amylase, and lipase showed no association with death.

Conclusion

LDH is a reliable prognostic marker for severe AP, correlating with severity indices and hospital stays. BISAP, CTSI, and MCTSI effectively predicted disease severity. NLR, LMR, and PLR may assist in monitoring recovery.

## Introduction

Acute pancreatitis (AP) is an inflammatory condition that affects the pancreas and potentially other organs [1]. It has an incidence of 35-80 cases per 100,000 individuals, with gallstones and alcohol being the leading culprits. Other contributors are hypertriglyceridemia, autoimmune diseases, trauma, and hereditary factors [2].

The 2012 Atlanta Classification divides AP into necrotizing and interstitial types based on the presence of necrosis [3]. AP has two phases: early, marked by systemic inflammatory response syndrome (SIRS), and late, which is characterized by local complications or persistent inflammation [4].

AP is classified as mild, moderately severe, or severe based on the presence of organ failure and complications. Patients with mild AP are characterized by limited local inflammation without any significant systemic inflammatory response. It is typically managed conservatively, and patients are often discharged within a week. Moderately severe AP is accompanied by local or systemic complications with or without transient organ failure (<48 hours) [5,6]. Severe AP is characterized by persistent single or multiple-organ failure, which carries a mortality rate of 20% to 40% [7].

Several classification systems have been established to assess the severity of AP. Commonly used methods include the Bedside Index of Severity in Acute Pancreatitis (BISAP), Ranson’s criteria, and the Acute Physiology and Chronic Health Evaluation (APACHE) II score. While these scoring systems are useful for determining disease severity, their reliability in predicting mortality remains limited [8].

There is increasing interest in rapid biomarkers for predicting prognosis in AP. Indicators, such as the neutrophil-lymphocyte ratio (NLR), lymphocyte-monocyte ratio (LMR), platelet-lymphocyte ratio (PLR), red cell distribution width (RDW), C-reactive protein (CRP), lactate dehydrogenase (LDH), and interleukin-6 (IL-6), have been studied for their association with severity and prognosis [9]. Among them, NLR within 48 hours is strongly associated with severe AP and functions as an independent adverse prognostic factor [10].

CT abdomen-based severity scoring helps in assessing the local complications, tissue damage, and pancreatic necrosis in AP. It aids in determining prognosis, guiding treatment decisions, and predicting outcomes [11].

Computed Tomography Severity Index (CTSI) and Modified CTSI (MCTSI) are two important radiological modalities to assess the severity of AP. CTSI (1990) categorizes severity as mild (scores 0-3), moderate (scores 4-6), or severe (scores 7-10) based on pancreatic inflammation and necrosis [12]. MCTSI (2004) refines this by including extra-pancreatic complications, classifying severity as mild (scores 0-2), moderate (scores 4-6), or severe (scores 8-10) for better prediction of outcomes [13].

This study was done to assess the prognostic value of various inflammatory markers and radiological and clinical severity indices in AP. This study explored the association between inflammatory markers, CTSIs, and clinical outcomes to identify a reliable, rapid, and cost-effective biomarker for predicting the prognosis of AP.

## Materials and methods

Study design and setting

This was a prospective observational study, conducted at All India Institute of Medical Sciences, Jodhpur, India, from January 2023 to July 2024. This study aimed to evaluate the prognostic value of inflammatory markers, including the NLR, LMR, RDW, CRP, IL-6, and LDH, in patients with AP. Secondary objectives included exploring the association between these inflammatory markers and the BISAP and the CTSI.

Severe acute pancreatitis (SAP) was defined according to the 2012 Revised Atlanta Classification, characterized by persistent organ failure lasting more than 48 hours [14]. Organ failure was assessed using the Modified Marshall Scoring System, which included respiratory failure, renal failure (defined as serum creatinine ≥ 1.9 mg/dL or the need for renal replacement therapy), and cardiovascular failure (systolic blood pressure < 90 mmHg) [15]. In this study, patients were divided into two groups based on organ failure and imaging findings suggestive of local complications, including peripancreatic fluid collections, pancreatic necrosis, pseudocysts, and walled-off necrosis. The first group was non-severe acute pancreatitis (N-SAP), which included mild cases. The second group was SAP, which included both moderately severe and severe cases.

Patient selection criteria

All patients with a diagnosis of AP were included in the study. The diagnosis of AP was confirmed based on the presence of an acute onset of characteristic abdominal pain with any one of the following criteria: (1) serum lipase or amylase levels three times the upper normal limit or (2) imaging findings consistent with AP on contrast-enhanced computed tomography (CECT) or ultrasound abdomen.

**Figure 1:**
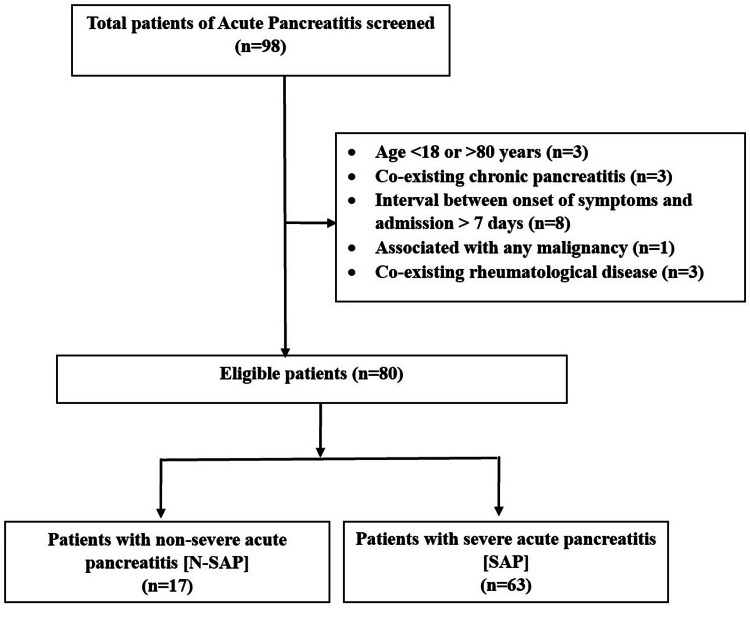
A flow chart of patients’ selection

The study excluded patients under 18 or over 80 years of age, those admitted more than seven days after symptom onset, individuals with severe trauma, or those pregnant. Patients with pre-existing chronic pancreatitis or pancreatic malignancy, other malignancies, and patients with rheumatological conditions were also excluded from this study (Figure 1).

Data collection

Patient data included demographics (age and gender) and underlying comorbidities such as diabetes mellitus and hypertriglyceridemia. Previous history of alcohol consumption and pancreatitis were recorded along with the BISAP score at the time of admission. Blood tests, including complete blood counts (CBC), amylase, lipase, CRP, LDH, LFT, RFT and IL-6 levels, were measured on the day of admission and again on day 7. A CT abdomen scan was done on the fifth day after the onset of the symptoms to assess the local complications and pancreatic necrosis. CT abdomen-based severity was evaluated using the CTSI developed by Balthazar et al. [12], and the MCTSI introduced by Mortele et al. [13]. Other parameters, such as the time from symptom onset to hospital admission and duration of hospital stay, were also noted. The NLR was determined by dividing the neutrophil count (10⁹/L) by the lymphocyte count (10⁹/L). The PLR was calculated as the platelet count (10⁹/L) divided by the lymphocyte count (10⁹/L). Similarly, the LMR was obtained by dividing the lymphocyte count (10⁹/L) by the monocyte count (10⁹/L) [9,10].

Statistical analysis

Data were analyzed using SPSS software (IBM SPSS Statistics for Windows, IBM Corp., Version 27, Armonk, NY). Continuous variables were expressed as mean ± standard deviation (SD) or median (interquartile range) and compared using the Student’s t-test or Mann-Whitney U test as appropriate. Categorical variables were presented as frequencies and percentages and analyzed using the chi-square or Fisher’s exact test. The correlation between severity markers and AP outcomes was assessed using Pearson’s or Spearman’s correlation coefficients. Receiver operating characteristic (ROC) curve analysis was performed to determine the predictive accuracy of LDH and BISAP for severe AP. A p-value < 0.05 was considered statistically significant.

## Results

Patient characteristics and clinical profile

In this study, 98 patients diagnosed with AP were initially screened. However, 18 patients were excluded based on the specified exclusion criteria. Consequently, 80 patients met the eligibility criteria for inclusion. The age distribution of enrolled patients ranged from 20 to 80 years, with a mean age of 40.9 ± 13.9 years. Most patients were male (60 out of 80, accounting for 75%), with a male-to-female ratio of 3:1. Among the male patients with AP, 39 (65%) were under the age of 40.

The observed etiological factors of AP include alcohol consumption in 41 patients (51%) and gallstones in 19 patients (24%), followed by hypertriglyceridemia in three patients (3.75%) and autoimmune pancreatitis in two patients (2.5%). The cause remained unidentified in 15 patients (19%). The most common presenting symptom of AP was abdominal pain (100%), followed by vomiting in 44 patients (55%) and abdominal distension in eight patients (10%). Other symptoms included shortness of breath, jaundice, and decreased urine output. The mean age of survivors was 40.7 ± 13.8 years, while the mean age of deceased patients was 46.7 ± 18.7 years (Table 1).

**Table 1:** Demographic characteristics, clinical findings, and outcomes in patients with acute pancreatitis (n = 80)

Characteristics	Number of cases (%)
Males	60 (75%)
Females	20 (25%)
M:F	3:1
Below 40 years	39 (65%)
Mean age in years (±SD)	40.9±13.9
Mean age for survived patients (years)	40.7±13.8
Mean age for deceased patients (years)	46.7±18.7
Etiology	
Gallstone disease	19 (24%)
Alcoholic	41 (51%)
Idiopathic/unknown	15 (19%)
Types of acute pancreatitis	
Mild pancreatitis	17 (21%)
Moderately severe pancreatitis	54 (68%)
Severe pancreatitis	9 (11%)
BISAP (Bedside Index for Severity in Acute Pancreatitis) Score	
Score 0-2 (mild pancreatitis)	64 (80%)
Score ≥3 (severe pancreatitis)	16 (20%)
Computed tomography abdomen findings	
Mildly bulky pancreas	8 (10%)
Interstitial edematous pancreatitis	21 (26%)
Necrotizing pancreatitis with peripancreatic collection/walled-off necrosis	49 (61%)
Computed Tomography Severity Index (CTSI)	
Mild (scores 0-3)	30 (37.5%)
Moderate (scores 4-6)	10 (12.5%)
Severe (scores 7-10)	40 (50%)
Modified CTSI (MCTSI)	
Mild (scores 0-2)	20 (25%)
Moderate (scores 4-6)	22 (27.5)
Severe (scores 8-10)	38 (47.5%)
Duration of hospital stay (days)	
0-10	32 (40%)
11-20	34 (42.5%)
>20	14 (17.5%)
Mortality	3 (3.75%)
Mortality in severe pancreatitis	3 out of 9 (33%)

**Figure 2:**
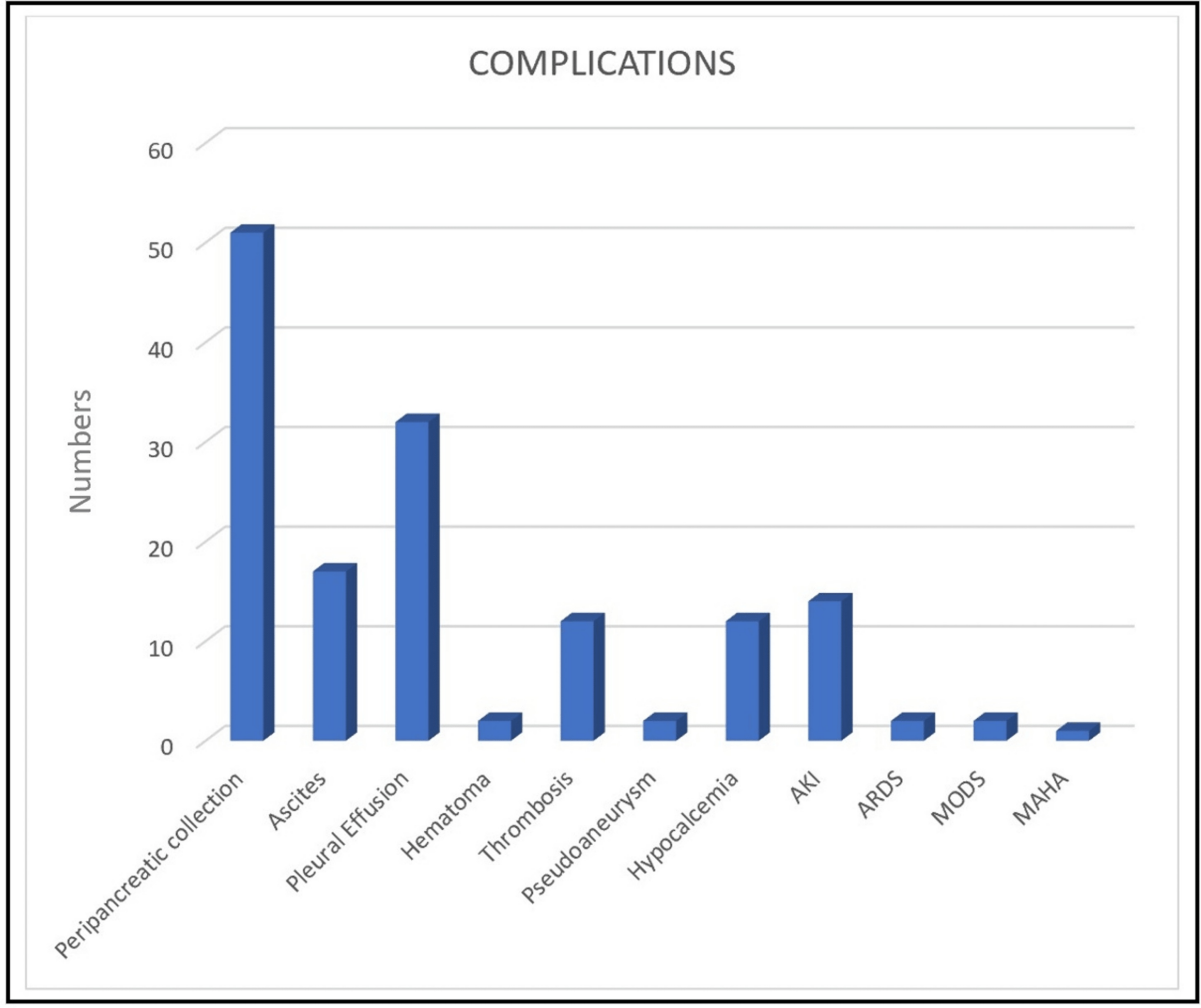
Local and systemic complications with their frequencies seen in patients of acute pancreatitis AKI - acute kidney injury, ARDS - acute respiratory distress syndrome, MAHA - microangiopathic hemolytic anemia, MODS - multiple organ dysfunction syndrome

Moderately severe pancreatitis was observed in 54 patients (68%), 17 (21%) had mild pancreatitis, and nine (11%) had severe pancreatitis. Complications were observed in 63 patients (79%), with the most common being peripancreatic collections in 49 patients (61%), pleural effusion in 31 patients (39%), ascites in 17 patients (21%), and acute kidney injury in 12 patients (15%) (Figure 2).

Severity and prognostic scoring

The severity of AP was evaluated using the CTSI and the MCTSI. Based on CTSI, 40 patients (50%) had severe pancreatitis, 10 (12.5%) had moderate, and 30 (37.5%) had mild pancreatitis. According to MCTSI, 38 patients (47.5%) had severe pancreatitis, 22 (27.5%) had moderate, and 20 (25%) had mild pancreatitis.

The BISAP score, which predicts the risk of complications and mortality, showed variability among patients. While 31% (25 patients) had a BISAP score of 0, indicating a lower risk, four patients had a BISAP score of 4. There were significant associations between BISAP, CTSI, and MCTSI scores with the severity of AP. BISAP demonstrated the strongest predictive value (OR 10.909, p < 0.0001), followed by MCTSI (OR 10.93, p = 0.008) and CTSI (OR 8.299, p = 0.025).

The BISAP score showed a strong association with mortality (χ² = 14.632, p < 0.001), while CTSI (χ² = 4.424, p = 0.035) and MCTSI (χ² = 4.519, p = 0.034) scores were also significantly associated with mortality.

Inflammatory markers and severity

Severe cases of acute pancreatitis had a lower mean age (38.9 ± 13.5 years) compared to non-severe cases (46.8 ± 13.8 years, p = 0.037). LDH was significantly higher in severe cases (527.5 ± 432.5 vs. 253.7 ± 126.7, p = 0.01). Urea levels were also elevated in severe cases (63.6 ± 50.1 vs. 25.8 ± 9.2, p = 0.003). Other inflammatory markers, including CRP, IL-6, ESR, and NLR, showed no statistically significant differences. In multivariate analysis, only serum LDH remained a statistically significant independent predictor of severe AP (p = 0.04). Age and urea levels were significantly associated with disease severity on univariate analysis, but they lost significance on multivariate analysis (p = 0.07 and p = 0.12, respectively), as mentioned in Table 2.

**Table 2:** Association of inflammatory markers and severity of acute pancreatitis (n = 80) CRP - C-reactive protein, ESR - erythrocyte sedimentation rate, IL-6 - interleukin-6, LDH - lactate dehydrogenase, LMR - lymphocyte-to-monocyte ratio, NLR - neutrophil-to-lymphocyte ratio, PLR - platelet-to-lymphocyte ratio, RDW - red cell distribution width

Markers	Non-Severe (n = 17) (mean ± SD)	Severe (n = 63) (mean ± SD)	p-value	t-value (where applicable)	Multivariate analysis (p-value)
Age (years)	46.82 ± 13.8	38.9 ± 13.5	0.03	-2.12	0.07
NLR	9.35 ± 4.4	11.56 ± 8.7	0.32	1.01	-
PLR	161.7 ± 91.6	226.5 ± 155.8	0.10	1.63	-
RDW	14.0 ± 0.6	14.5 ± 1.4	0.12	1.55	-
CRP	104.7 ± 73.1	128.4 ± 86.1	0.30	1.04	-
ESR	36 ± 25	42 ± 30	0.49	0.69	-
LDH	253.7 ± 126.7	527.5 ± 432.5	0.01	2.57	0.04
Urea	25.8 ± 9.2	63.6 ± 50.1	0.003	3.08	0.12
IL-6, median (IQR)	38.8 (12-63)	61 (32.6-107.1)	0.09	-	-
Amylase, median (IQR)	518 (416-682)	617 (305-1197)	0.5	-	-
Lipase, median (IQR)	560 (446-1030)	928 (364-2370)	0.27	-	-

Significant changes were observed in inflammatory and pancreatic biomarkers from baseline to day 7. LDH levels decreased significantly (p = 0.02), and serum amylase and lipase levels also showed marked reductions (Table 3).

**Table 3:** Comparison of baseline and day 7 biomarker levels in patients with acute pancreatitis ^*^One patient expired on day 5 of admission. ^#^t-value is applicable for parametric data (paired t-test), and z-value is applicable for non-parametric data (Wilcoxon test for paired samples). CRP - C-reactive protein, ESR - erythrocyte sedimentation rate, IL-6 - interleukin-6, LDH - lactate dehydrogenase, NLR - neutrophil-to-lymphocyte ratio

	Baseline biochemical values (mean ± SD)/median (IQR) on the day of presentation (n = 80)	(Mean ± SD)/Median (IQR) on day 7 of admission (n = 79*)	p-value	t-value/z- value^#^
CRP (mg/L)	125.7 ± 83	113.6 ± 68.2	0.14	1.48
ESR (mm/hour)	40.9 ± 29.3	44.8 ± 32.3	0.24	-1.16
NLR	11.7 ± 8.2	7.9 ± 5.9	0.001	3.97
LDH (IU/L)	467.8 ± 403.1	359.7 ± 227.7	0.02	3.23
IL-6 (pg/mL), median (IQR)	58.1 (29.6-89.5)	56.1 (19.7-125)	0.30	1.96
Amylase (IU/L), median (IQR)	659.5 (345.2-1196.2)	68 (43.2-117.5)	0.01	7.77
Lipase (IU/L), median (IQR)	1072.5 (442.2-2329.7)	99 (66-194.5)	0.01	7.76

Prognostic significance of serum LDH

Serum LDH levels were significantly higher in patients with a BISAP score ≥2 compared to those with a score <2 (mean LDH: 897 U/L vs. 351 U/L, p < 0.0001). Similarly, LDH levels were markedly different in patients with an MCTSI score <6 vs. those with a score ≥6 (mean LDH: 320 U/L vs. 630 U/L, p < 0.0001).

**Figure 3:**
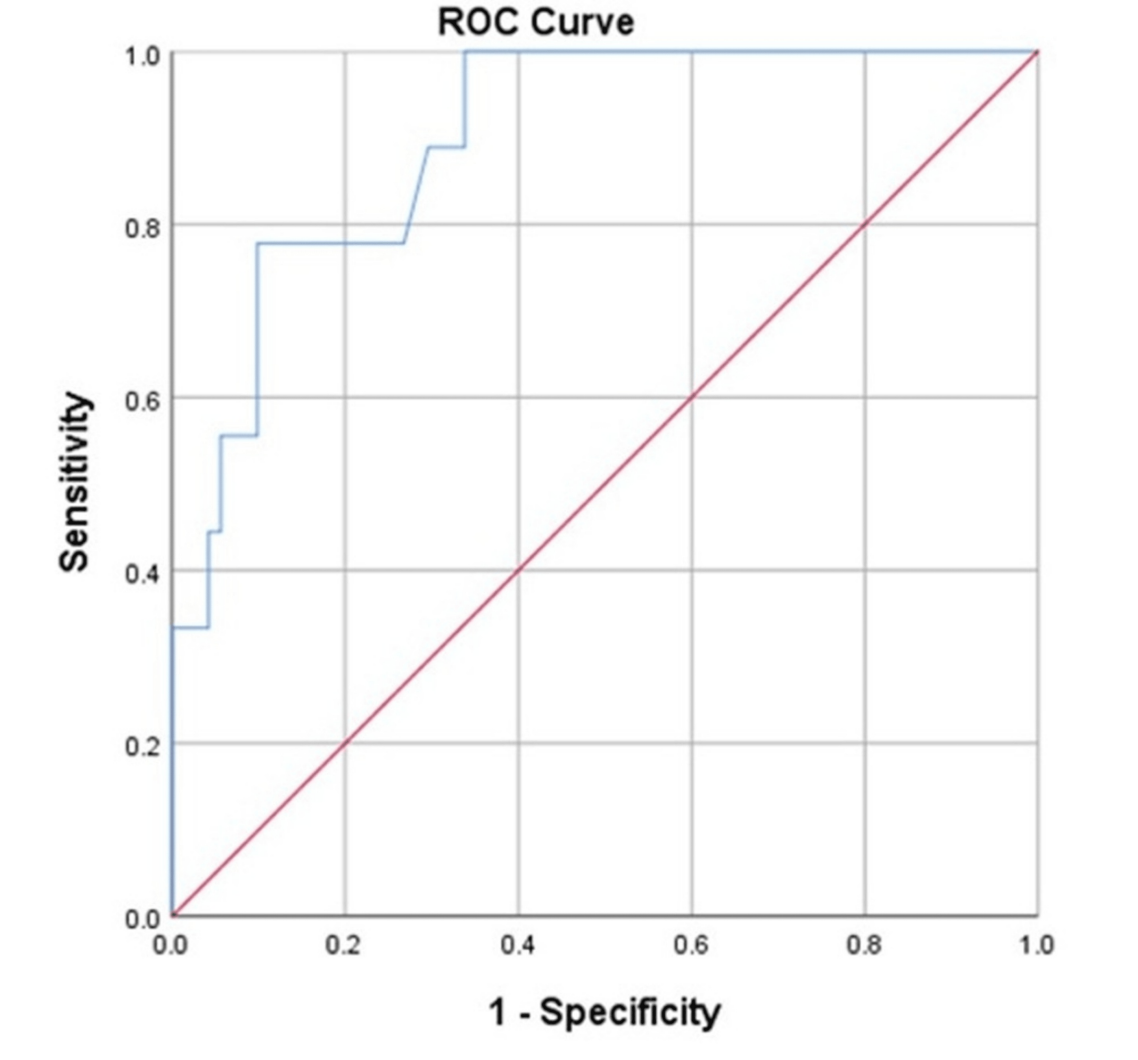
ROC curve of LDH for predicting severe acute pancreatitis The area under the curve, with a 95% confidence interval of 0.898 (0.810-0.987) and a p-value of <0.0001. Serum LDH of >435.00 has 100% sensitivity and 66.8% specificity to predict the severity of acute pancreatitis. LDH - lactate dehydrogenase, ROC - receiver operating characteristic

Serum LDH level >435 U/L demonstrated 100% sensitivity and 66.8% specificity in predicting the severity of AP (AUC = 0.898; 95% CI: 0.810-0.987; p < 0.0001) (Figure 3).

Clinical outcome

All patients received aggressive intravenous fluids, except those with cardiovascular comorbidities, along with analgesics, antiemetics, and symptomatic management. Antibiotics were administered only in cases of proven infected necrosis or acute necrotizing pancreatitis with severe sepsis. Forty-four patients (55%) required antibiotic therapy along with supportive care. Among the 80 patients, three succumbed during hospitalization, resulting in a mortality rate of 3.75%.

## Discussion

The term "pancreatitis" refers to an acute or chronic inflammation of the pancreas that is characterized by premature activation of digestive enzymes within the pancreatic acinar cells, resulting in pancreatic auto-digestion [16].

In our study of 80 patients, males were predominantly affected, with a male-to-female ratio of 3:1, with a mean age of 40.9 ± 13.9 years. Pain in the abdomen, vomiting, and abdominal distension were the most common symptoms. Additional symptoms observed included shortness of breath, jaundice, and decreased urine output.

In our study, alcohol (51%) and gallstones (24%) were the primary causes of AP. This finding aligns with the observations of Weiss et al. [17], who reported that these two factors were responsible for 70% of AP. Other etiologies included hypertriglyceridemia and autoimmune pancreatitis. In our study, alcohol-related pancreatitis was more frequent among males, whereas gallstone-related pancreatitis was predominantly seen in females. These findings are consistent with the observations reported by Lankisch et al. [18].

The BISAP score was evaluated for all patients on the day of admission. Among the 80 patients studied, 64 (80%) had a score of 0 or 1, while 16 (20%) had a score of 2 or higher. A BISAP score between 0 and 2 was indicative of a low mortality risk (less than 2%), whereas scores ranging from 3 to 5 were associated with a significantly higher mortality risk (greater than 15%).

Zhou et al. [19], in their study on severity stratification and prognostic prediction of patients with AP at the early phase, suggested that BISAP was the single most valuable predictor for SAP. In our study, the BISAP score predicted the severity of pancreatitis. The odds ratio of BISAP for having severe pancreatitis was 10.909 (95% CI: 2.368- 50.266) (p < 0.0001). BISAP could not predict local complications.

Consistent with Sahu et al. [20] and Banday et al. [21], our study demonstrated a strong correlation between CTSI and MCTSI scores with the severity of pancreatitis and its associated complications.

NLR, PLR, and LMR showed no significant differences between severe and non-severe pancreatitis. They also showed no significant correlation with CTSI, MCTSI, or BISAP scores. They did not predict severity or complications, nor correlate with hospital stay or severity scores. Our findings differ from those of Liu et al. [22], who reported significantly higher NLR and PLR in severe AP.

However, a significant reduction in NLR levels (p = 0.001) was observed by day 7 as compared to baseline. NLR may be useful for monitoring the disease progression and recovery rather than as a severity predictor.

In this study, elevated serum LDH levels were associated with higher BISAP, CTSI, and MCTSI scores, as well as prolonged hospital stays. In our study, LDH levels were significantly higher in severe pancreatitis. The mean LDH in severe cases was 527.5 ± 432.5. In non-severe cases, the value was considerably lower at 253.7 ± 126.7. Chen et al. [23] also found elevated LDH levels in severe pancreatitis. They reported mean values of 476 U/L in severe and 287 U/L in non-severe cases. Their results are comparable to our findings.

Higher serum LDH levels are significantly associated with increased severity of AP. LDH > 435 U/L accurately predicts severe cases, while >274 U/L helps to identify the patients at higher risk of complications. Our results align with the study by Huang et al. [24], which found that serum LDH levels above 290 U/L were associated with increased AP severity scores, longer hospital stays, higher medical expenses, and increased mortality. 

Serum LDH levels reflect underlying tissue damage and necrosis. It may help improve prognostic accuracy when combined with the BISAP score. When CT imaging is unavailable or delayed, elevated LDH can serve as an alternative marker to identify patients at higher risk who may need urgent referral, closer monitoring, or early intervention.

Higher BUN levels have been linked to worse outcomes in AP. Studies show that elevated BUN predicts mortality, multiorgan failure, and severity [25,26]. In the current study, mean blood urea was significantly higher in severe (63.6 mg/dL) than non-severe cases (25.8 mg/dL) (p < 0.0001), reinforcing its prognostic value. Serum urea levels also showed a significant association with mortality.

Sathyanarayan et al. [27] conducted a study on 108 patients with AP. Their findings revealed that IL-6 levels on day 3 were significantly elevated in severe cases (p = 0.04). However, in the present study, IL-6 levels did not show a significant association with disease severity. In our study, IL-6 was assessed on the day of presentation and day 7 of admission, by which time cytokine levels may have declined, potentially missing their peak predictive value observed during the early inflammatory phase (typically within 48-72 hours).

As noted previously, the average mortality rate in AP is approximately 2%-10% [28]. In our study, three out of 80 patients died during hospitalization, resulting in a mortality rate of 3.75%. Hidalgo et al. [29] reported a similar mortality rate in their study, supporting our observation. However, Jain et al. [30] reported a higher mortality rate; their Indian study showed an 8% mortality rate. This difference may be attributed to variations in the severity of AP at admission, referral bias, and differences in the study population.

This study was limited by its single-center design and relatively small sample size, which may affect the generalizability of the results. As a leading tertiary care center of western Rajasthan (India), our institution receives a higher proportion of severe pancreatitis cases. This referral bias may overestimate disease severity trends. Additionally, long-term follow-up was not performed, limiting assessment of delayed outcomes.

## Conclusions

This study assessed prognostic biomarkers in AP, identifying LDH as the most reliable indicator of severity and complications, with a strong correlation to hospital stay duration. It provides clinicians with accessible and cost-effective tools for the early identification of high-risk AP cases. LDH and BISAP scores serve as effective risk stratification measures. Elevated LDH (>435 U/L) and BUN indicate severe disease, aiding in early referral and intensive monitoring, optimizing triage, resource allocation, and timely specialist intervention.
